# A Cerium Organic Framework with {Cu_2_I_2_} Cluster and {Cu_2_I_2_}*_n_* Chain Modules: Structure and Fluorescence Sensing Properties

**DOI:** 10.3390/s23052420

**Published:** 2023-02-22

**Authors:** Bin Tan, Zi-Wei Li, Zhao-Feng Wu, Xiao-Ying Huang

**Affiliations:** 1 Fujian Science & Technology Innovation Laboratory for Optoelectronic Information of China, Fuzhou 350108, China; 2 State Key Laboratory of Structural Chemistry, Fujian Institute of Research on the Structure of Matter, The Chinese Academy of Sciences, Fuzhou 350002, China

**Keywords:** copper iodine, coordination polymer, near infrared fluorescence, biothiol, sensing

## Abstract

In this work, a copper iodine module bearing a coordination polymer (CP) with a formula of [(Cu_2_I_2_)_2_Ce_2_(INA)_6_(DMF)_3_]·DMF (**1**, HINA = isonicotinic acid, DMF = *N*,*N’*-dimethyl formamide) is presented. The title compound features a three dimensional (3D) structure, in which the {Cu_2_I_2_} cluster and {Cu_2_I_2_}*_n_* chain modules are coordinated by N atoms from a pyridine ring in INA^−^ ligands, while the Ce^3+^ ions are bridged by the carboxylic groups of INA^−^ ligands. More importantly, compound **1** exhibits an uncommon red fluorescence (FL) with a single emission band maximized at 650 nm belonging to near infrared (NIR) luminescence. The temperature dependent FL measurement was applied to investigate the FL mechanism. Remarkably, **1** could be used as a FL sensor to cysteine and the nitro-bearing explosive molecule of trinitropheno (TNP) with high sensitivity, demonstrating its potential FL sensing applications for biothiol and explosive molecules.

## 1. Introduction

Fluorescent coordination polymers (FL-CPs) have been widely developed in the past two decades, not only due to their colorful luminescence but also their potential applications in solid state lighting [1,2,3,4,5], sensing [6,7,8,9,10,11], bioimaging [11,12,13,14,15], and so forth. Thus far, most of the reported FL-CPs exhibit luminescence within the visible light spectrum. Comparatively, the FL-CPs with near infrared (NIR) emission are highly desirable because of their promising applications in biological fields e.g., biosensing, bioimaging, and photothermal therapy [16,17,18,19]. However, NIR emitting CPs are still rare at present. In the limited reported NIR FL-CPs, rare earth (RE) metals are usually used, while it is still a challenging task to seek appropriate organic linkers to sensitize RE centered emission [20]. Recently, the NIR FL-CPs were successfully assembled from the fluorescent organic linkers with emission in the NIR region [21,22]. Recently, CuI bearing CPs have received intense attention due to their tunable fluorescence by monitoring organic ligands (e.g., derivates of pyridine, imidazole, triazole) that coordinated to the inorganic cluster and chain-like CuI modules [23,24,25,26,27,28,29]. Through deliberately selecting unique N-bearing ligands to tune the electronic band structure of the CuI modules by coordination, NIR emitting could be achieved in CuI hybrids [23,26]. However, designing and synthesizing organic linkers with NIR emitting chromophores also requires complex operations. Therefore, developing new NIR FL-CPs which could be economically and conveniently prepared is desirable.

On the other hand, thiol molecules have played a crucial role in natural and physiological systems [30,31,32,33]. The concentration changes in cellular thiols even with a very small range of fluctuation can induce a number of diseases. The leaking of thiols and their derivatives can also cause safety and environment issues [34]. Many conventional physicochemical methods, e.g., high-performance liquid chromatography and electrospray ionization-mass spectrometry, have been commonly used to monitor the thiol species [35]. However, expensive instrumentation and the tedious technically demanding steps to some extent hinder their applications especially in the undeveloped areas. Consequently, development of efficient and economical fluorescent probes with high selectivity and sensitivity towards thiol molecules is highly demanded [36,37,38,39]. To date, compared with organic fluorescent sensors, FL-CP probes for thiol molecules are still limited [40,41,42,43]. As far as we are aware, the NIR FL CP sensor to thiols has never been reported.

**Scheme 1:**
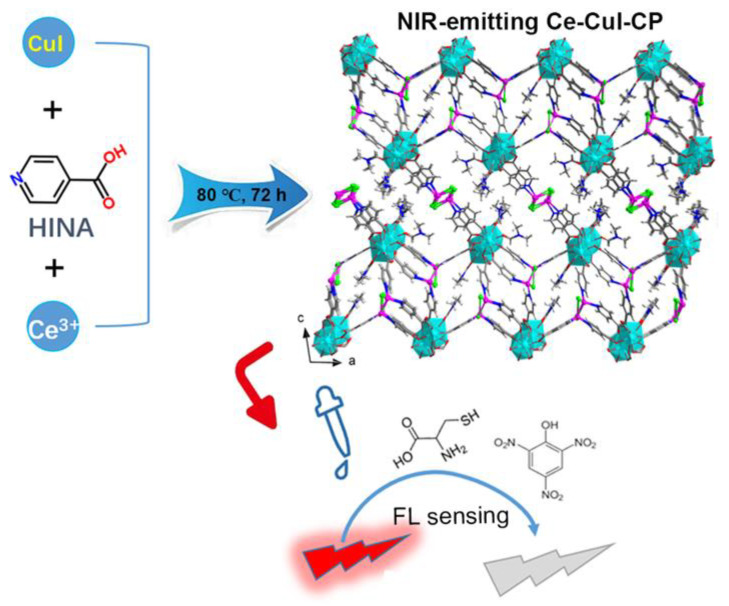
Schematic illustration of the synthesis of compound **1** with NIR fluorescence and luminescence sensing performance towards Cys.

Herein, a NIR emitting CP formulated as [(Cu_2_I_2_)_2_Ce_2_(INA)_6_(DMF)_3_]·DMF (**1**, HINA = isonicotinic acid, DMF = *N*,*N*’-dimethyl formamide) is synthesized and characterized. The compound features a three-dimensional (3D) framework (Scheme 1). Remarkably, **1** exhibits an uncommon copper iodine centered emission maximized at 650 nm ascribed to the NIR emitting when excited under 465 nm light. The temperature dependent FL was measured to study the emitting mechanism. More importantly, due to the specific affinity between the cysteine and inorganic CuI emitting center, **1** exhibits a selective FL quenching behavior to cysteine (Cys). Moreover, **1** shows FL sensing performance to explosive molecule of trinitrophenol (TNP). This work would provide a good reference on designation of novel NIR emitting FL CPs.

## 2. Materials and Methods

Reagents: Ce(NO_3_)_6_·6H_2_O (≥ 99%, Adamas-beta); KI and CuI (99%, Adamas-beta); cysteine (97%, Adamas-beta); DMF (*N*,*N*’-dimethyl-formamide), CH_3_CN and ethanol (AR, Greagent). All the reagents above are purchased from Shanghai Titan Chemical Co., Ltd., Shanghai, China; TNP (AR) was purchased from Sinopharm Chemical Reagent Co., Ltd., Shanghai, China).

Preparation of **1**: A mixture of 100 mg Ce(NO_3_)_6_·6H_2_O, 100 mg CuI, 150 mg KI, 100 mg HINA, 3.0 mL DMF, 2.0 mL CH_3_CN and 2.0 mL ethanol was sealed in a glass vessel, heated at 80 °C for 3 days, and then cooled to room temperature. The light red clavate crystals were selected by hand, washed with absolute ethanol, and then dried in the air (80 mg, 33.65% yield based on cerium, Figure S1). Elemental analyses calculated for **1**: C 26.58%, N 6.06%, H 2.13% were found to be: C 26.74%, N 6.32%, H 2.64%.

Physical measurements: Powder X-ray diffraction (PXRD) patterns were recorded on a Rigaku MiniFlex II diffractometer using CuK*α* radiation (λ = 1.54178 Å). A graphite monochromator was used and the generator power settings were set at 44 kV and 40 mA. Data were collected between 2*θ* of 3–50° with a scanning speed of 1.0°/min (Figure S2). Thermogravimetric (TG) data were collected on a TA Q50 Analyzer with a temperature ramping rate of 10 °C/min from 30 to 700 °C under nitrogen gas flow (Figure S3). Single crystal X-ray diffraction data were collected with graphite-monochromated CuK*α* (*λ* = 1.54178 Å) using a SuperNova diffractometer. The Multi-temperature FL spectra of **1** at the solid state were performed on a FLS1000 spectrometer while FL detection were recorded using a PerkinElmer LS55 FL spectrometer. Infrared spectra were measured in the range of 4000–300 cm^−1^ using KBr pellets on a Nicolet iS10 FT-IR spectrometer (Figure S2).

FL measurements: The as-made crystalline samples of **1** were manually ground to obtain fine powders. Then 2 mg powdered samples were dispersed in 2 mL either organic solvents or ethanol solutions of 10^−3^ M cysteine and TNP, which were ultrasonicated for several minutes to get a stable emulsion. The suspension was placed in a quartz cell of 1 cm width for FL measurement. The FL sensing experiments were conducted by preparing ultrasonication induced suspensions of 2 mg compound **1** in 2 mL ethanol, and 10^−3^ M cysteine or TNP with various concentrations was injected to the resulting suspensions by using a pipette. For all the measurements, the dispersed emulsions of compound **1** were excited at 465 nm while the corresponding emission wavelengths were monitored from 580 nm to 800 nm.

X-ray crystallography: A single crystal of compound **1** suitable for single crystal X-ray diffraction (SCXRD) was selected under an optical microscope and glued to a thin glass fiber. The structure was solved by direct methods and refined with full-matrix least squares techniques using the *SHELX*2018 package [44]. The lattice DMF molecule is highly disordered and thus was not identified from the difference-Fourier maps; instead, its contribution to the overall intensity data was treated using the SQUEEZE method in PLATON [45]. The CCDC number is 2233846 for compound **1**. The detailed crystallographic data and structure-refinement parameters are listed in Table 1.

## 3. Results and Discussion

### 3.1. Crystal Structure Analysis

**Figure 1:**
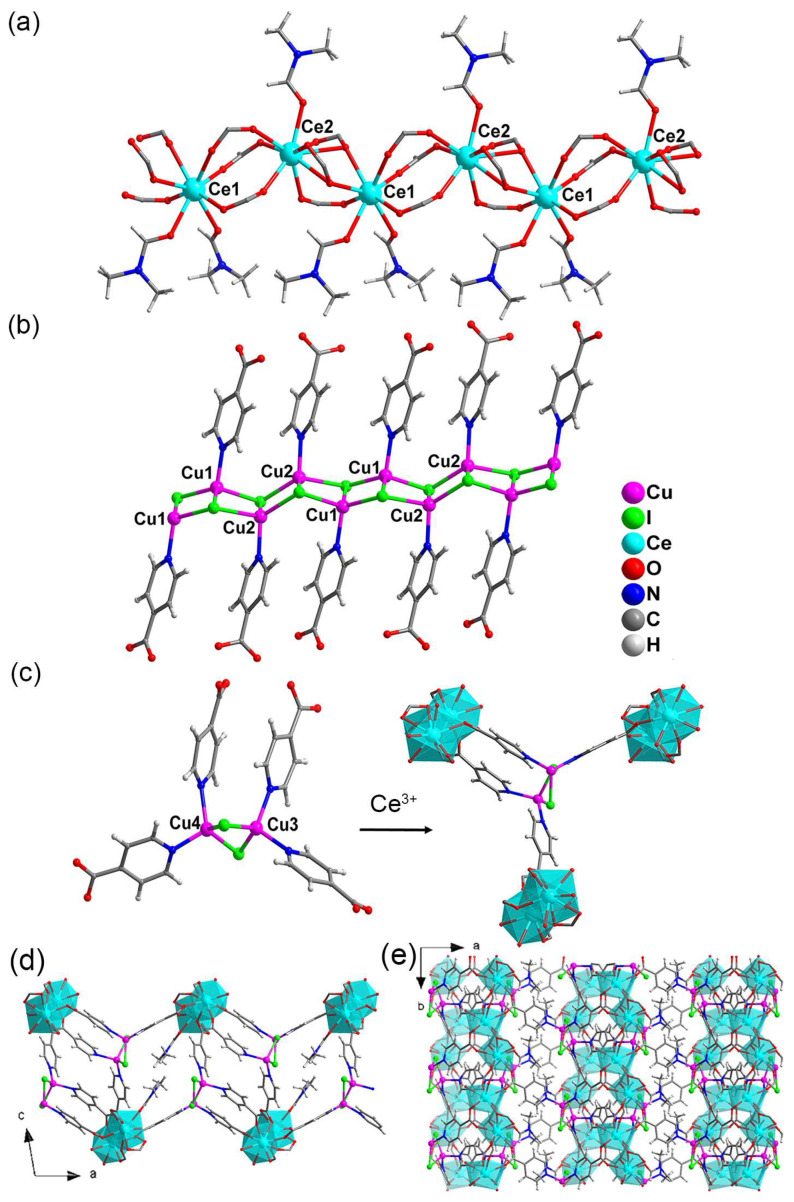
(**a**) The 1 D {-Ce-(COO)_6_(DMF)_3_-}*_n_* chain with coordination environment of Ce^3+^ ion in compound **1**. (**b**,**c**) are the coordination environment of the copper iodine modules. The coordination environments for Ce^3+^ in (**c**) are highlighted in polyhedral mode. (**d**,**e**) are the 2D layer forming by Ce^3+^ and the {Cu_2_I_2_} bearing tetradentate carboxylic ligand viewed from the *b* and *c* axis, respectively.

High quality crystals of compound **1** with high yield were obtained by the assembly of HINA with CuI and Ce^3+^ ions via a solvothermal method (Scheme 1). Single crystal diffraction analysis indicated the title compound exhibits a three-dimensional (3D) structure. The asymmetric unit contains two crystallographically independent Ce^3+^ ions, two {Cu_2_I_2_} units, six INA^−^ ligands, three terminal DMF molecules, and one lattice DMF molecule. In compound **1**, the INA^−^ ligand plays a dual-coordinating role, that is, the carboxylic group and pyridine in INA^−^ ligand are coordinated to Ce^3+^ ions and copper iodine modules, respectively. The Ce(1) atom is eight coordinated, in which the six carboxylic groups adopt monodentate coordinating way and the last two sites are occupied by terminal DMF molecules, Figure 1a. The Ce(2) atom is nine coordinated by six carboxylic groups from six different INA^−^ ligands, and two of which adopt chelating and four take a monodentate coordinating mode, respectively; the DMF molecule acting as terminal ligand occupies the last coordination site. The neighboring Ce(1) and Ce(2) atoms are bridged by sharing one monodentate and two chelating coordinating carboxylic groups to generate a 1D {-Ce_2_-(COO)_6_(DMF)_3_-}_n_ chains (Figure 1a). As shown in Table 2, the Ce-O bond values varied from 2.358(9) to 2.831(10) Å, which is consistent with that in former literature [46,47,48,49].

The inorganic CuI modules in **1** exhibit two different structure features. As depicted in Figure 1b, Cu(1) and Cu(2) are interconnected by I atoms to form a 1D ladder like inorganic {Cu_2_I_2_}_n_ belt. The INA^−^ ligands are anchored on such 1D inorganic chains by Cu-N coordination to form a unique 1D {Cu_2_I_2_(INA)_2_}_n_ ligands (Figure 1b). The Cu(3) and Cu(4) modules are coordinated by two I atoms to form a {Cu_2_I_2_} dimer, in which the Cu atoms are further bridged by N atoms from four different INA^−^ ligands to generate a tetradentate carboxylic ligand (Figure 1c). The coordination bond lengths for Cu-I and Cu-N are locating in 2.592(2)~2.764(3) Å and 2.032(11)~2.106(12) Å, respectively, which are also consistent with that in the former literature [50,51,52,53,54] (Table 2). The resultant {Cu_2_I_2_} bearing ligands bridge the 1D {-Ce_2_-(COO)_6_(DMF)_3_-}_n_ chains to form a 2D layered structure (Figure 1d,e). Then, such 2D layers are bridged by the Figure 1b depicted {CuI-INA}_n_ modules as pillar linkers to form a 3D structure (Figure S3). The disordered and coordinated DMF molecules occupy the 1D channels viewed along the b axis and (111) direction, respectively (Figure S3).

### 3.2. Basic Measurements

**Figure 2:**
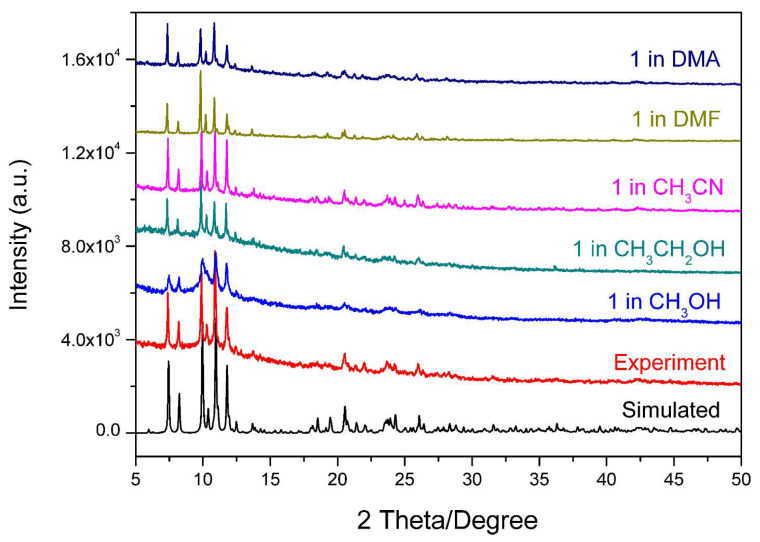
The PXRD pattern of the as-made **1** and samples immersed in solvents over 24 h compared with the simulated data.

**Figure 3:**
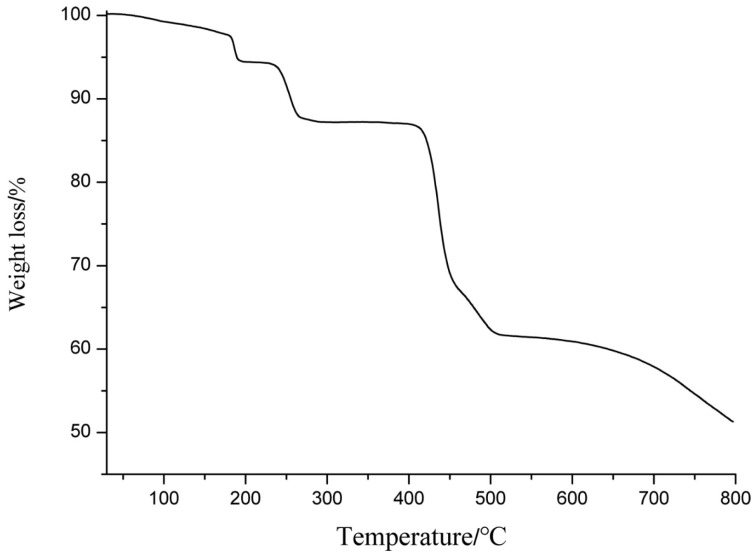
The TG curve for the as-made compound **1**.

The purity of the compound was identified by PXRD measurement. As depicted in Figure 2, the experiment PXRD pattern was almost the same as that calculated from the crystal data, demonstrating the as-made crystalline sample was a purity phase. The PXRD patterns of the samples that were immersed in common lab used solvents were still the same as that of the as-made sample, demonstrating the structure of **1** was stable in organic solvents (Figure 2). The thermal stability of the as-made compound was also investigated. Thermogravimetric (TG) analysis indicated that compound **1** started to lose one free DMF molecule at around 185 °C, corresponding to the first weight loss of 3.63% (Calc. 3.58%). Then the second step of mass loss of 6.97% was observed at 245 °C corresponding to the removal of three coordinated DMF molecules (Calc. 7.06%). After removing the coordinated DMF, the weight was nearly unchanged until 400 °C, then a steep sudden drop was observed corresponding to the collapse of the structure (Figure 3).

### 3.3. Photoluminescence Studies

**Figure 4:**
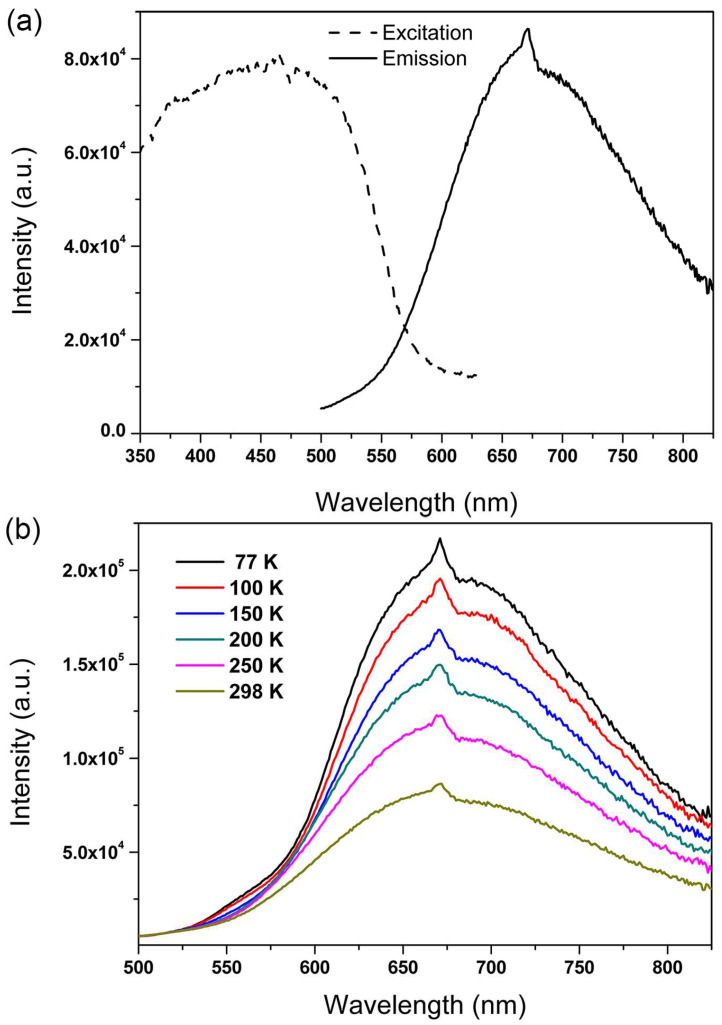
(**a**) The solid-state FL spectra of the as-made **1** measured at room temperature. (**b**) Emission spectra of **1** recorded at various temperatures excited at 465 nm.

The solid-state FL of the as-made compound was measured at room temperature. As shown in Figure 4a, compound **1** exhibited an uncommon NIR FL maximized at 650 nm when excited by 465 nm light. The chromaticity coordinate was (0.40, 0.59) that was located at the value of the red-light region (Figure S4). The free HINA ligand had no FL whether the excitation wavelength was monitored or the width of FL instrument slit was increased to the maximized value (Figure S5). The FL of Ce^3+^ ion usually arises from the 4f−5d transition and the 5d orbitals are very sensitive to the ligand sphere [55,56,57]. Therefore, the nonluminous HINA is not a good sensitizer to generate Ce^3+^ centered characteristic FL. The luminescence of **1** was similar to that of other copper iodide CPs and can be assigned to a copper iodine-centered excited state deriving from halide-to-metal charge transfer. The temperature dependent FL of **1** as measured varied from 77 K to 298 K. As shown in Figure 4b, as temperature decreased, the FL intensity of **1** increased gradually. The phenomenon could be ascribed to the decrease of thermally induced nonradiative-decay pathways. This result is consistent with that the former copper iodine-based hybrids with thermally activated delayed FL, suggesting the emission of **1** at room temperature originates from the inorganic copper iodine modules [58,59,60,61].

**Figure 5:**
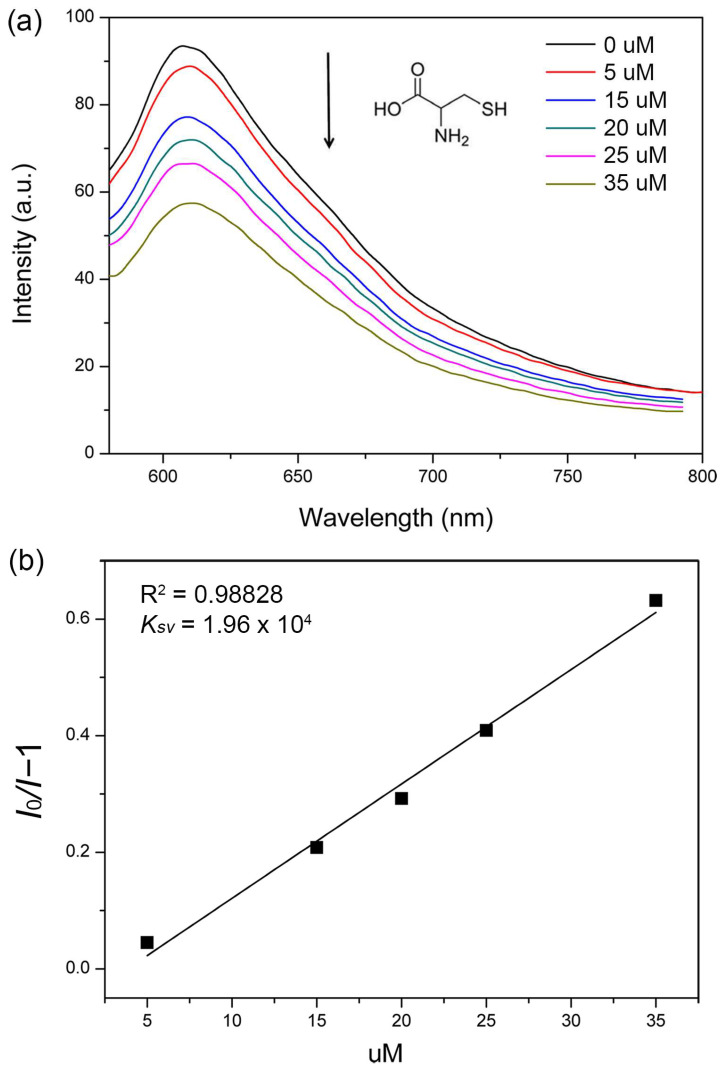
(**a**) FL spectra of **1** upon addition of various amounts of 10^−3^ M Cys. The excitation wavelength was 465 nm. (**b**) The *K*_sv_ curve for the quenching of **1** by 10^−3^ M Cys.

**Figure 6:**
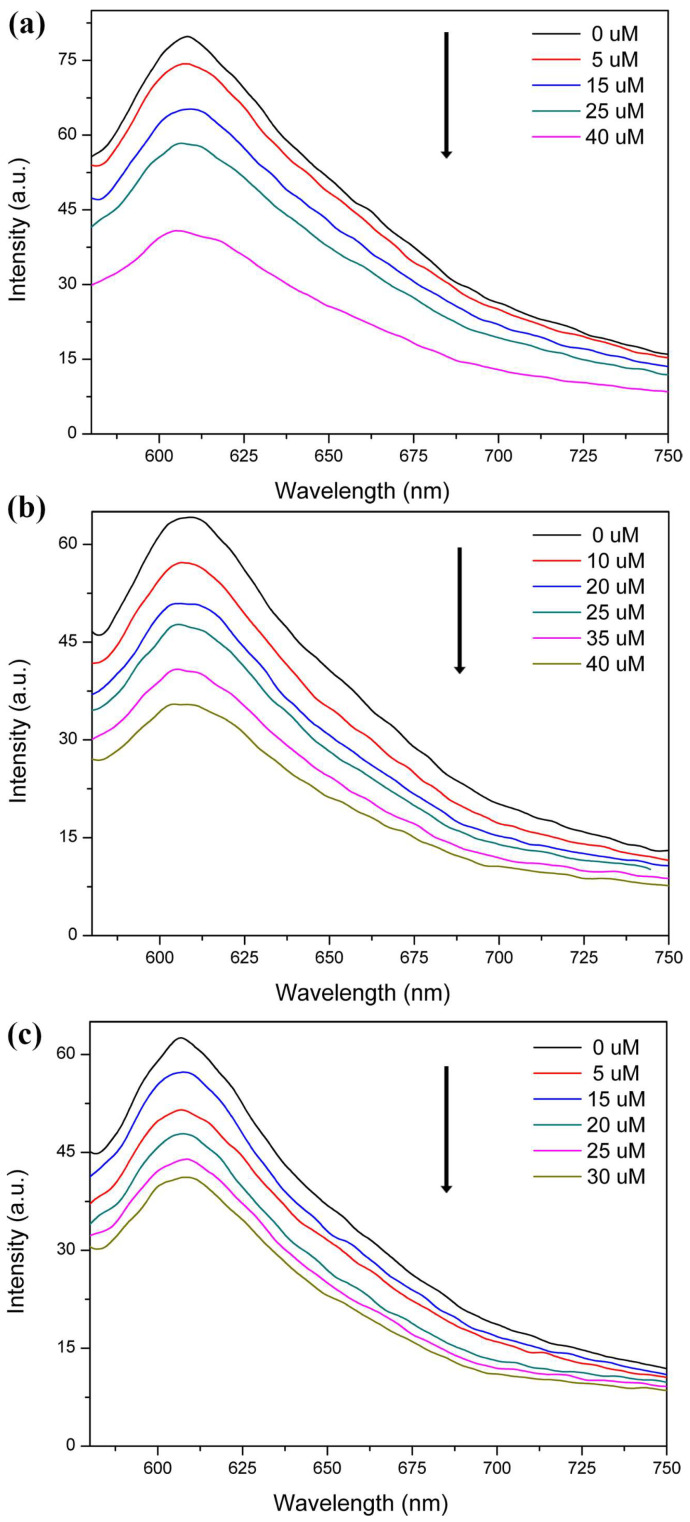
FL spectra of **1** dispersed in 10^−3^ M Na^+^ (**a**), Ca^2+^ (**b**) and Cl^−^ (**c**) upon addition of various amounts of 10^−3^ M Cys. The excitation wavelength was 465 nm.

**Figure 7:**
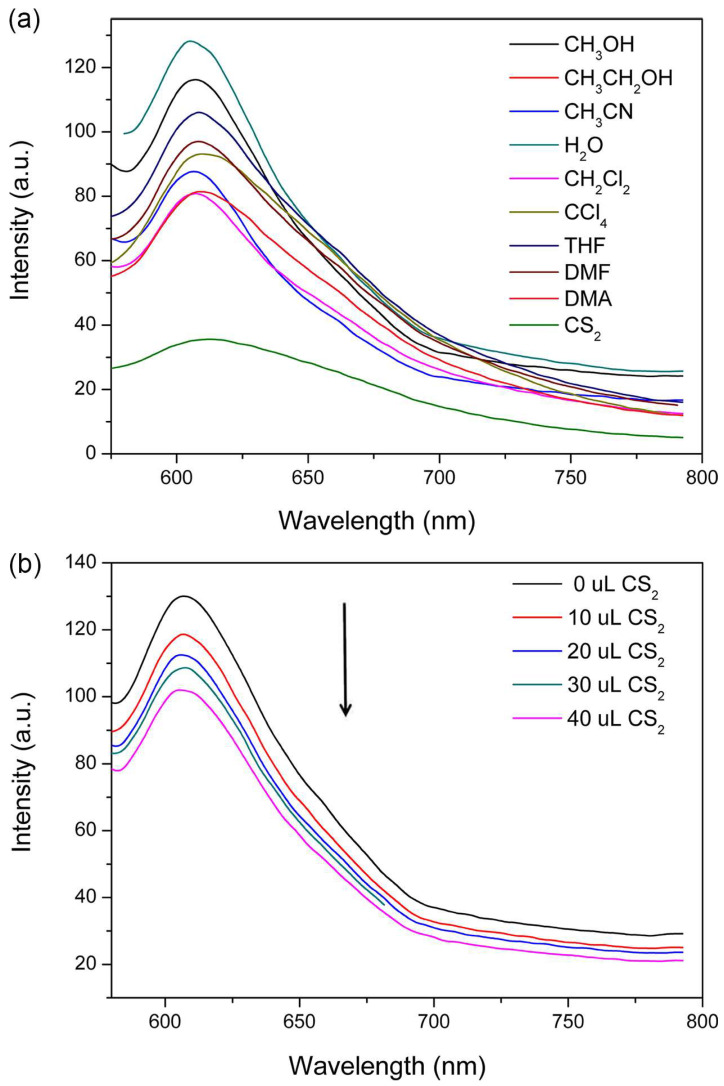
(**a**) The FL spectra of **1** dispersed in different solvents. (**b**) The FL spectra of **1** with the addition of CS_2_. The excitation wavelength was 465 nm.

The copper atom usually has good affinity towards sulfydryl group bearing molecules based on the hard and soft acids and bases theory. Therefore, Cys acting as a representative of bio-thiols was used as an analyst to explore the potential sensing property of **1**. First, 2 mg of powdered **1** was dispersed in 2 mL ethanol and the solution of 10^−3^ M Cys was added to monitor the FL response. As depicted in Figure 5a, the FL signal quenched gradually as a function of increasing Cys concentration, and by more than 50% when as little as 35 μM Cys was added. This result demonstrates that compound **1** could act as a potential NIR FL sensor to thiol molecules especially in a biosystem. To quantitatively analyze the quenching effect, the SV equation of *I*_0_/*I* = 1 + *K*_sv_[M] (*I_0_* and *I* are the suspension luminescence intensity of compound **1** without and with addition of Cys, respectively, and [M] is the molarity of Cys and *K*_sv_ is the quenching constant) was analyzed [62,63]. As seen in Figure 5b, the SV plot displays a good linear behavior and the *K*_sv_ was calculated to be 1.96 × 10^4^ M^−^. Using the *K*_sv_ value as a reference, the detection sensitivity of compound **1** is better than that of other CP-complex sensors [42,43]. As we know, Cys usually coexists with metal ions and anions in biological environment. Therefore, the sensing performance of **1** dispersed in the 10^−3^ M representative Na^+^, Ca^2+^ and Cl^−^ ethanol solutions was also detected. As depicted in Figure 6, the FL intensity of **1** in Na^+^, Ca^2+^ and Cl^−^ solutions exhibited a sensitive quenching response to Cys as that without interferential metal ions and anions, demonstrating the good sensing selectivity of **1** for probing of Cys. In order to further demonstrate the selective detecting performance to S-bearing molecules, CS_2_, the regular but toxic lab-used solvent, was used to further check the sensing selectivity of **l**. As depicted in Figure 7a, although the FL of compound **1** exhibited various degree of FL intensity changes to various common lab used solvents, the most obvious FL quenching response was observed in the case of CS_2_ (Figure 7b). The FL intensity was decreased by adding the CS_2_ into the FL emulsion of compound **1**, indicating its detecting selectivity to S-bearing molecules.

**Figure 8:**
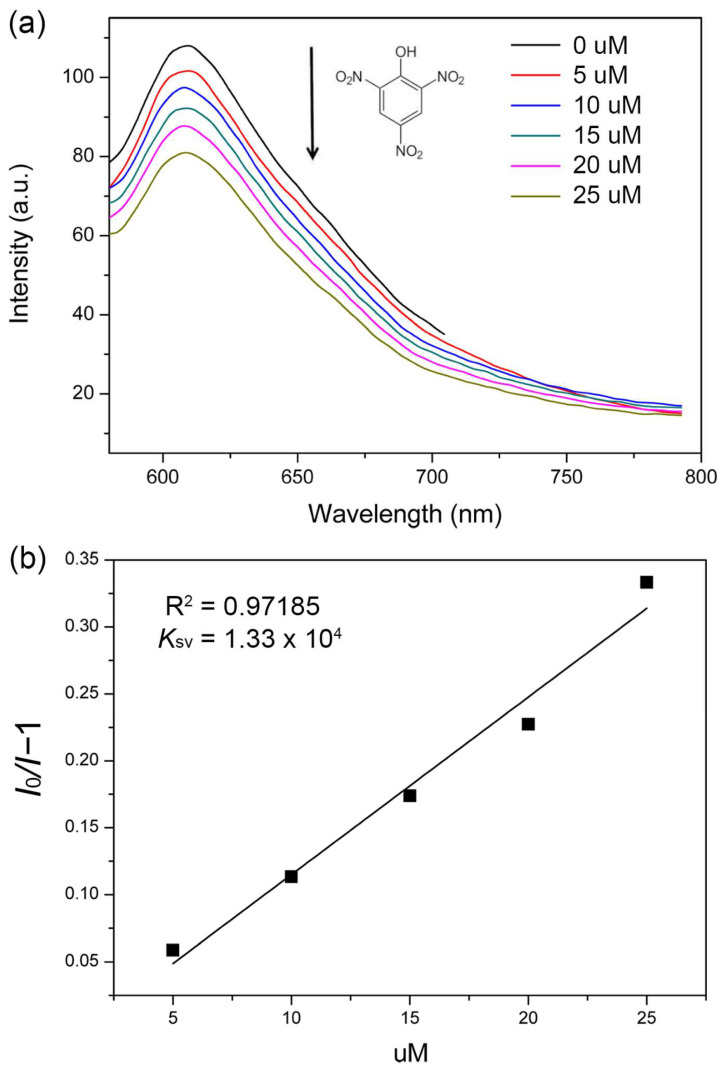
(**a**) FL spectra of **1** upon addition of various amounts of 10^−3^ M TNP. (**b**) The *K*_sv_ curve for the quenching of **1** by 10^−3^ M TNP.

The polynitro compounds are important explosive materials which are widely used in military and industrial applications. Therefore, developing convenient FL sensors to detect nitro explosives is of great importance to life health and nation safety. Herein, compound **1** also shows a FL quenching effect to TNP molecule. As seen in Figure 8a, the FL intensity is quenched gradually by adding 10^−3^ M ethanol solution of TNP into the emulsion of **1**. The SV plot exhibits a good linear relationship and the *K*sv value was 1.33 × 10^4^ M^−^ (Figure 8b), which is comparable to the other reported CP sensors based on the representative transition and rare earth metal ions (Table 3) [64,65,66,67,68,69]. The FL emulsion of **1** dispersed in other lab used solvent molecules e.g., DMF, methanol and CH_3_CN also show the sensitive FL quenching behavior as that dispersed in ethanol, indicating the sensing selectivity towards TNP (Figure S6a–c). The compound could be reused at least for five times which still showed selective detection performance for TNP molecule (Figure S6d). As we know, the TNP with three nitro groups has strong electron withdrawing ability, then the excited electrons of **1** is transferred to TNP molecules which caused the quenching of luminescence [7].

## 4. Conclusions

In conclusion, we presented and characterized a 3D Ce-CP bearing inorganic copper iodine module. The compound exhibited a NIR FL, indicating that confinement of emitting copper iodine modules into CPs would be an alternative way to design NIR emitting materials. Importantly, the compound could be used for FL detection of Cys and TNP with sensitivity and selectivity. This work would provide a good reference to developing NIR FL materials and sensors with promising applications in biological fields.

## Figures and Tables

**Table 1:** Crystallographic data and structural refinement detail for the title sample.

Empirical formula	C_48_H_52_Ce_2_Cu_4_I_4_N_10_O_16_
Formula weight	2066.99
Crystal system	monoclinic
Space group	*C*2/*c*
*T*/K	298(2)
*λ*/Å	1.54178
*a*/Å	30.1437(5)
*b*/Å	8.55370(10)
*c*/Å	51.9807(9)
*α*/º	90
*β*/º	100.926(2)
*γ*/º	90
*V*/Å^3^	13159.8(4)
*Z*	8
*D_c_*/Mg·m^−3^	2.087
*μ*/mm^−1^	27.175
*F*(000)	7856
Measured refls.	58,283
Independent refls.	11,566
*R*_int_	0.1042
No. of parameters	712
*GOF*	1.058
*^a^R*_1_, *^b^wR*_2_ [*I* > 2*σ*(*I*)]	0.0735, 0.1854
*^a^R*_1_, *^b^wR*_2_ (all data)	0.0830, 0.1931

*^a^R*_1_ = ∑║*F*_o_│−│*F*_c_║/∑│*F*_o_│. *^b^wR*_2_ = {∑*w*[(*F*_o_)^2^−(*F*_c_^2^)]^2^/∑*w*(*F*_o_^2^)^2^}^1/2^.

**Table 2:** The selected bond values (Å) for compound **1**.

**Ce-O bond values**			
Ce(1)-O(1)	2.509(9)	Ce(1)-O(3)#1	2.502(8)
Ce(1)-O(5)#4	2.435(8)	Ce(1)-O(7)#5	2.506(8)
Ce(1)-O(9)#6	2.565(8)	Ce(1)-O(12)#3	2.358(9)
Ce(1)-O(13)	2.538(11)	Ce(1)-O(14)	2.528(10)
Ce(2)-O(1)	2.831(10)	Ce(2)-O(2)	2.525(8)
Ce(2)-O(4)#2	2.468(9)	Ce(2)-O(6)	2.452(8)
Ce(2)-O(8)#5	2.436(8)	Ce(2)-O(9)#6	2.706(8)
Ce(2)-O(10)#6	2.589(9)	Ce(2)-O(11)#5	2.424(9)
Ce(2)-O(15)	2.476(9)		
**Cu-I bond values**			
Cu(1)-I(1)	2.626(2)	Cu(1)-I(1)#1	2.628(2)
Cu(1)-I(2)	2.737(3)	Cu(2)-I(1)	2.764(3)
Cu(2)-I(2)	2.635(3)	Cu(2)-I(2)#2	2.624(3)
Cu(3)-I(3)	2.674(2)	Cu(3)-I(4)	2.633(2)
Cu(4)-I(3)	2.592(2)	Cu(4)-I(4)	2.760(2)
**Cu-N bond values (Å)**			
Cu(1)-N(1)	2.044(10)	Cu(2)-N(2)	2.050(11)
Cu(3)-N(3)	2.071(11)	Cu(3)-N(4)	2.058(10)
Cu(4)-N(5)	2.032(11)	Cu(4)-N(6)	2.106(12)

Symmetry transformations used to generate equivalent atoms: #1 −x+1/2, −y+5/2, −z+1; #2 −x+1/2, −y+3/2, −z+1; #3 x+1/2, y+3/2, z; #4 x, y+1, z; #5 x+1/2, y+1/2, z; #6 −x+1/2, y+3/2, −z+1/2.

**Table 3:** Summary of reported CP-based FL sensors for TNP.

Compound	Detection Media	*K*_sv_ Value/M^−^	Reference
Compound 1	ethanol	1.33 × 10^4^	This work
Cd(INA)(pytpy)(OH)·2H_2_O	DMF	4.3 × 10^4^	[64]
[Mg_2_Zn_2_(OH)_2_(NDC)_3_(H_2_O)_2_]·6H_2_O	ethanol	0.94 × 10^4^	[65]
UiO-68@NH_2_	H_2_O	5.8 × 10^4^	[66]
[L_4_Cd_3_(H_2_O)_2_]	H_2_O	6.6 × 10^4^	[67]
[NH_2_(CH_3_)_2_][Zn_4_O(bpt)_2_(bdc-NH_2_)_0.5_]·5DMF	DMF	1.69 × 10^4^	[68]
[NH_2_(CH_3_)_2_][Zn_4_O(bpt)_2_(bdc-NH_2_)_0.5_]·5DMF		6.19 × 10^4^	
[Tb_2_(PBA)_3_(H_2_O)_3_]·DMF·3H_2_O	H_2_O	4.5 × 10^4^	[69]

## Data Availability

Not applicable.

## Supplementary Materials

The following supporting information can be downloaded at https://www.mdpi.com/article/10.3390/s23052420/s1, Figure S1: The photograph of the as-made compound 1; Figure S2: The IR spectra of compound 1; Figure S3: The 3D structure of 1 viewed at different directions; Figure S4: The photograph of the CIE chromaticity diagram for 1; Figure S5: The solid state FL spectra of the free HINA ligand measured at room temperature; Figure S6: The FL sensing spectra of 1 towards 10^−3^ M TNP when dispersed in varied lab solvents and the cycling sensing performance of 1.
