# Supplementary material for: A Cerium Organic Framework with {Cu2I2} Cluster and {Cu2I2}n Chain Modules: Structure and Fluorescence Sensing Properties
Source: Sensors (Basel). 2023 Feb 22;23(5):2420. doi: 10.3390/s23052420 (PMC10007347; doi:10.3390/s23052420)
Supplement: Supplementary file 1 [file sensors-23-02420-s001.zip › sensors-2180929-supplementary.pdf]

A cerium organic framework with {Cu_2I_2} cluster and {Cu_2I_2}$_n$ chain modules: structure and fluorescence sensing properties

Bin Tan [1,2], Zi-Wei Li [1,2], Zhao-Feng Wu [1,2] and Xiao-Ying Huang [1,2,*]

[1] Fujian Science & Technology Innovation Laboratory for Optoelectronic Information of China, Fuzhou, Fujian 350108, P. R. China; tanbin060412@163.com;

[2] State Key Laboratory of Structural Chemistry, Fujian Institute of Research on the Structure of Matter, the Chinese Academy of Sciences, Fuzhou, Fujian, 350002, P.R. China; liziwei@fjirsm.ac.cn.

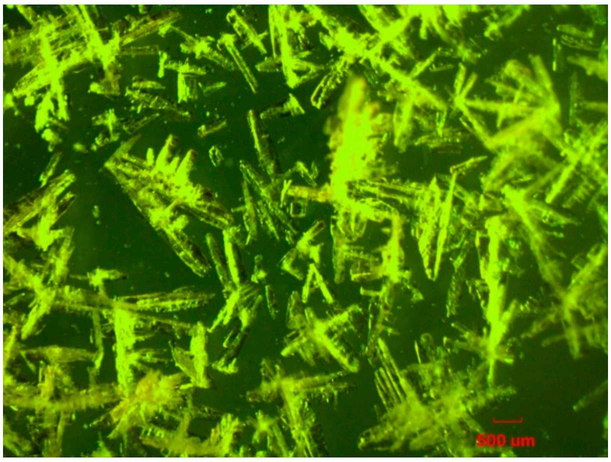

Figure S1. The photograph of the as-made compound **1**.

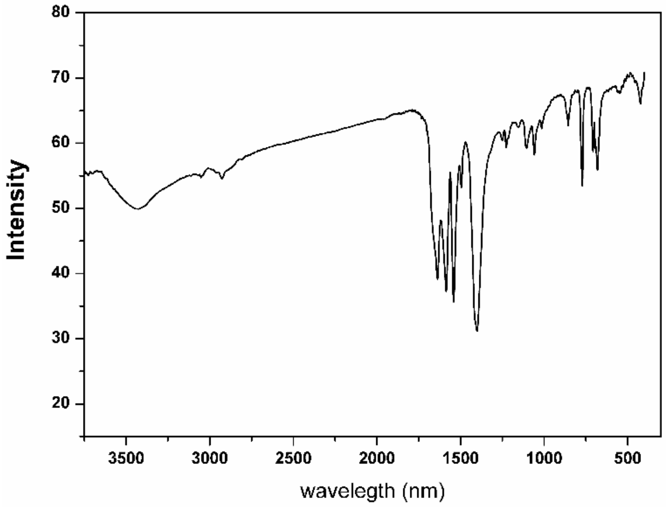

Figure S2. The IR spectra of compound **1**.

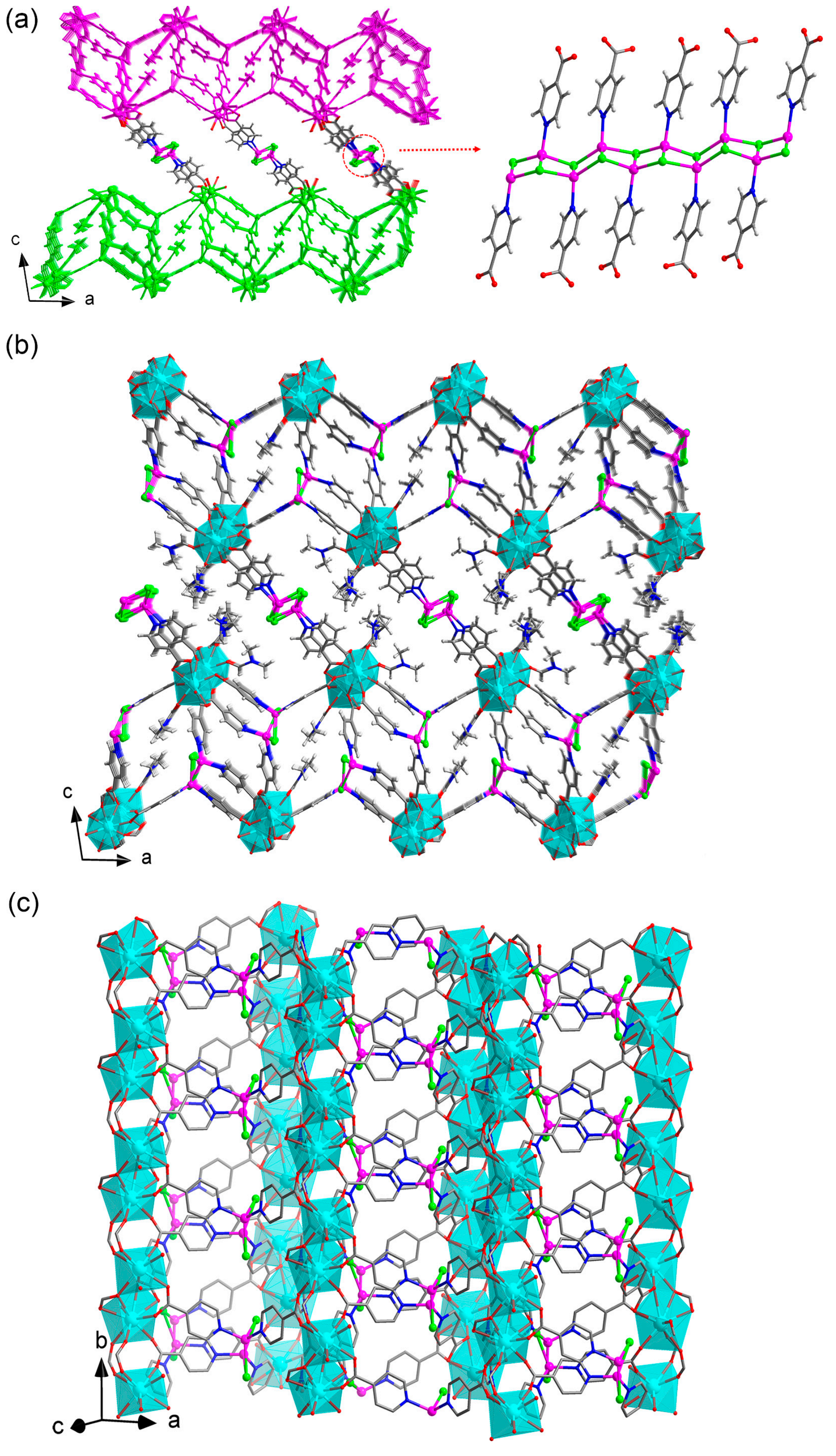

Figure S3. (a) The 3D structure of **1** formed by bridging the 2D layer with the 1D pillared CuI-INA ligands. For clarity, the neighboring layered structures are highlighted by rose red and green color, and the disordered DMF were removed between the layers. (b) and (c) are the 3D structure of **1** viewed from the *b* axis and [111] direction, respectively. The hydrogen atoms in Figure 2c have been omitted for clarity.

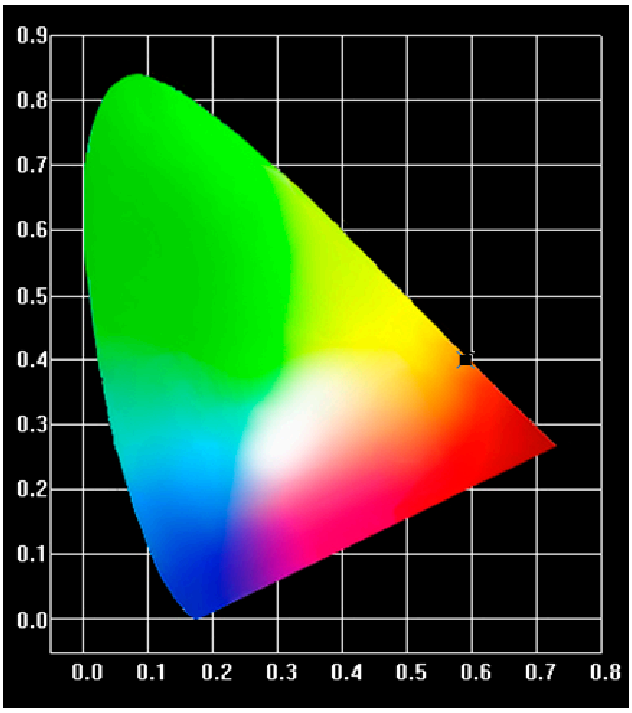

Figure S4. The photograph of the CIE chromaticity diagram for **1**.

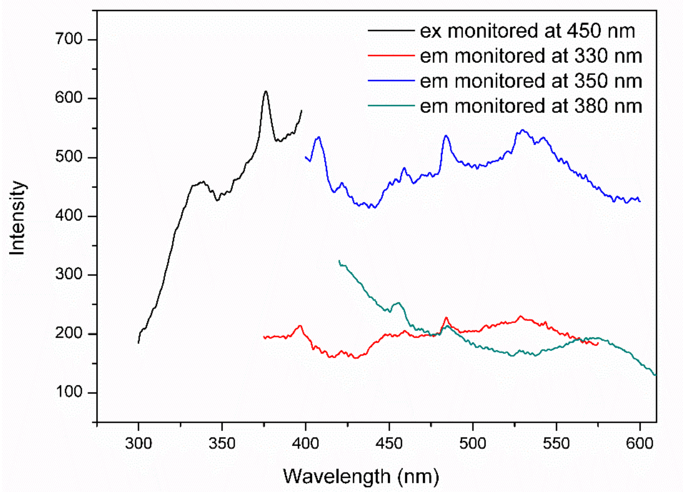

Figure S5. The solid state FL spectra of the free HINA ligand measured at room temperature.

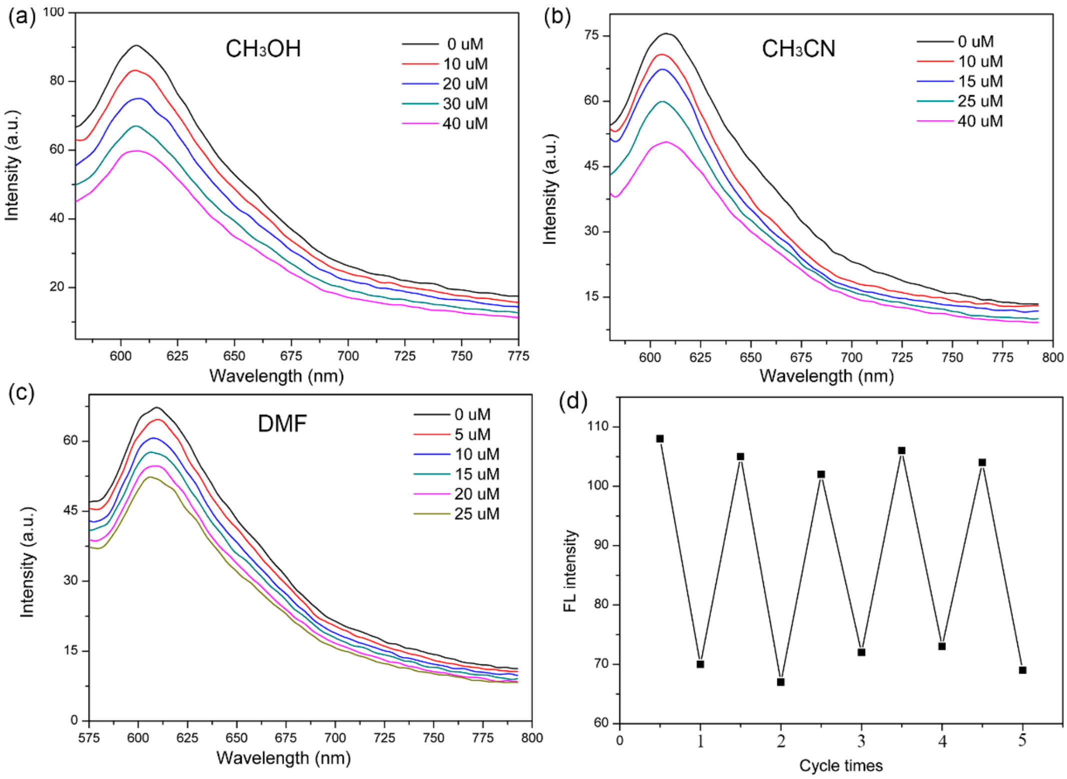

Figure S6. FL spectra of **1** dispersed in CH_3OH (a), CH_3CN (b) and DMF (c) upon addition of various amounts of 10^{-3} M TNP. (d) The cycling sensing performance of **1** by recording FL intensity with and without 30 μM TNP solution.
